# Landscape of kidney replacement therapy provision in low- and lower-middle income countries: A multinational study from the ISN-GKHA

**DOI:** 10.1371/journal.pgph.0003979

**Published:** 2024-12-02

**Authors:** Victoria Nkunu, Somkanya Tungsanga, Hassane M. Diongole, Abdulshahid Sarki, Silvia Arruebo, Fergus J. Caskey, Sandrine Damster, Jo-Ann Donner, Vivekanand Jha, Adeera Levin, Masaomi Nangaku, Syed Saad, Feng Ye, Ikechi G. Okpechi, Aminu K. Bello, David W. Johnson, Marcello Tonelli

**Affiliations:** 1 Division of General Internal Medicine, Faculty of Medicine and Dentistry, University of Alberta, Edmonton, Alberta, Canada; 2 Division of Nephrology and Immunology, Faculty of Medicine and Dentistry, University of Alberta, Edmonton, Alberta, Canada; 3 Division of General Internal Medicine-Nephrology, Department of Medicine, Faculty of Medicine, Chulalongkorn University, Bangkok, Thailand; 4 Division of Nephrology, Department of Medicine, National Hospital Zinder, Zinder, Niger; 5 Faculty of Health Sciences, University of Zinder, Zinder, Niger; 6 Nephrology Unit, National Hospital Abuja, Abuja, Federal Capital Territory, Nigeria; 7 The International Society of Nephrology, Brussels, Belgium; 8 Population Health Sciences, Bristol Medical School, University of Bristol, Bristol, United Kingdom; 9 George Institute for Global Health, University of New South Wales (UNSW), New Delhi, India; 10 School of Public Health, Imperial College, London, United Kingdom; 11 Manipal Academy of Higher Education, Manipal, India; 12 Division of Nephrology, Department of Medicine, University of British Columbia, Vancouver, British Columbia, Canada; 13 Division of Nephrology and Endocrinology, The University of Tokyo Graduate School of Medicine, Tokyo, Japan; 14 Division of Nephrology and Hypertension, University of Cape Town, Cape Town, South Africa; 15 Kidney and Hypertension Research Unit, University of Cape Town, Cape Town, South Africa; 16 Department of Kidney and Transplant Services, Princess Alexandra Hospital, Brisbane, Queensland, Australia; 17 Centre for Kidney Disease Research, University of Queensland at Princess Alexandra Hospital, Brisbane, Queensland, Australia; 18 Translational Research Institute, Brisbane, Queensland, Australia; 19 Australasian Kidney Trials Network at the University of Queensland, Brisbane, Queensland, Australia; 20 Department of Medicine, University of Calgary, Calgary, Alberta, Canada; 21 Canada and Pan-American Health Organization, World Health Organization’s Collaborating Centre in Prevention and Control of Chronic Kidney Disease, University of Calgary, Calgary, Alberta, Canada; Sheffield Hallam University, UNITED KINGDOM OF GREAT BRITAIN AND NORTHERN IRELAND

## Abstract

In low- and lower-middle-income countries (LLMICs), delivering equitable kidney care presents substantial challenges, resulting in significant disparities in disease management and treatment outcomes for people with kidney failure. This comprehensive report leveraged data from the International Society of Nephrology-Global Kidney Health Atlas (ISN-GKHA), to provide a detailed update on the landscape of kidney replacement therapy (KRT) in LLMICs. Among the 65 participating LLMICs, reimbursement for KRT (publicly funded by the government and free at the point of delivery) was available in 28%, 15%, and 8% for hemodialysis (HD), peritoneal dialysis (PD), and kidney transplantation (KT), respectively. Additionally, while 56% and 28% of LLMICs reported the capacity to provide quality HD and PD, only 41% reported accessibility to chronic dialysis, defined as >50% of the national population being able to access KRT, and a mere 5% LLMICs reported accessibility to KT. Workforce shortages in nephrology further compound these challenges. Kidney registries and comprehensive policies for non-communicable diseases and chronic kidney disease care were limited in LLMICs. A comprehensive and cost-effective approach is crucial to address these challenges. Collaboration at global, regional, country, and individual levels is essential to enhance the quality of kidney care across LLMICs.

## Introduction

Chronic kidney disease (CKD), particularly when it advances to kidney failure, is a significant global health burden [1]. The difficulties posed by kidney failure are more complex to manage in settings with limited resources [2, 3]. Low-income (LICs) and lower-middle-income (LMICs) countries (LLMICs) face challenges in providing equitable kidney care, leading to disparities in disease management, quality of care, treatment outcomes, and overall patient well-being [4]. These disparities stem from fundamental limitations in the kidney health system infrastructure across LLMICs. Constraints on healthcare expenditure and resource allocation impede access to kidney replacement therapy (KRT), including hemodialysis (HD), peritoneal dialysis (PD), and kidney transplantation (KT), leaving many people struggling to receive essential care [5]. Moreover, these healthcare systems often lack comprehensive kidney registries, reliable data, and sufficient resources, including trained personnel and specialized equipment [6, 7]. The inadequate availability and capacity of services compromise the overall quality of kidney care in LLMICs.

Recognizing the urgent need to address these inequities, the International Society of Nephrology Global Kidney Health Atlas (ISN-GKHA) Project was launched as a comprehensive, multinational survey to map the existing capacity of kidney care systems across different world regions. This manuscript leverages data from the ISN-GKHA to describe the organization, processes, and structures for the care of people with kidney failure living in LLMICs [8]. We aimed to descriptively compare the findings from LLMICs with those from high-income countries (HICs) to highlight the areas that required targeted interventions, aiming to elevate the quality of global kidney care.

## Materials and methods

The ISN-GKHA employed two strategies to gather data at a country level. The first part was from an in-depth literature review of published data, grey literature, kidney registries, and databases to find the prevalence of CKD, incidence and prevalence of treated kidney failure, cost of KRT, and healthcare budgets. The second part was based on a multinational survey. The detailed methods have been previously published [8, 9].

### Survey development, validation, and administration

The survey questionnaire underwent development and validation through consultations with relevant experts, the ISN Executive Committee, and ISN regional leaders. Administered as an online questionnaire through REDCap (www.project-redcap.org), the survey was distributed between June 1 and September 30, 2022 via the ISN’s 10 regional boards, encompassing the following regions: Africa, Eastern and Central Europe, Latin America, the Middle East, North America and the Caribbean, North and East Asia, Oceania and South East Asia (OSEA), Newly Independent States (NIS) and Russia, South Asia, and Western Europe.

The ISN-GKHA working group, in collaboration with ISN regional boards and national leaders, conducted thorough follow-ups via email and telephone to secure comprehensive survey responses. In each participating country, we included at least three key stakeholders knowledgeable about the status and scope of national kidney care practices. These stakeholders included a nephrology society leader, a healthcare policymaker, and an advocate from a patient representative organization.

### Data handling and reporting

Responses from surveys conducted in French and Spanish were translated into English by certified translators. To maintain data accuracy, internal validation of the responses was carried out by each key stakeholder. External validation was then performed by ISN regional leaders, who were consulted to evaluate the consistency and quality of the compiled data. Any discrepancies identified during the review process were systematically addressed through follow-up inquiries with survey stakeholders. Further validation at national and regional levels included cross-referencing the findings with published literature, government reports, and input from survey respondents. The data from individual surveys were extracted and cleaned using Microsoft Excel. Subsequently, the cleaned data were consolidated into a unified database, securely maintained in a computer system with automated backup protocols.

### Definitions of key variables

The key variables examined various facets of KRT and related factors. These included an assessment of funding and cost of KRT, gaps in total and government health budgets, the prevalence of treated KRT, and the availability of centers for provision of KRT. The accessibility to KRT was evaluated based on whether >50% of the national population could access KRT. The assessment of the capacity of quality KRT involved the availability of adequate HD frequency, focusing on the capacity for a 3-4-hour HD session thrice weekly. For PD, the assessment considered the capacity for four daily exchanges on continuous ambulatory PD (CAPD) or equivalent cycles on automated PD. Additionally, the study explored health information systems (HD, PD, and KT registries) as well as policy and advocacy elements, including the availability of non-communicable diseases (NCDs), CKD-specific policies, and the recognition of KRT as a health priority.

### Data analysis

The statistical analysis was performed utilizing STATA 17 software (Stata Corporation, 2017). The analysis was conducted at the country level, and the outcomes were presented descriptively, including counts with percentages or medians with interquartile ranges (IQR), as appropriate. Results were stratified based on the ISN region [10] and the World Bank income group, determined by the Gross National Income (GNI) per capita [11] as of April 2022. The findings in LICs and LMICs were compared to those in HICs as a standard reference for an ideal setting.

### Ethics approval

The University of Alberta Research Ethics Committee approved this project (protocol number: PRO00063121). Consent was obtained by email from survey respondents. Our study did not report experiments on humans and/or the use of human tissue samples.

## Results

### Survey response

Of 167 countries which provided survey responses to the ISN-GKHA, 65 were LLMICs, comprising 20 (31%) LICs and 45 (69%) LMICs. The participating countries were distributed across various ISN regions, including Africa (n = 36), OSEA (n = 10), South Asia (n = 7), Latin America (n = 4), the Middle East (n = 4), and NIS and Russia (n = 4) (S1 Table).

### Funding and cost of KRT

**Fig 1:**
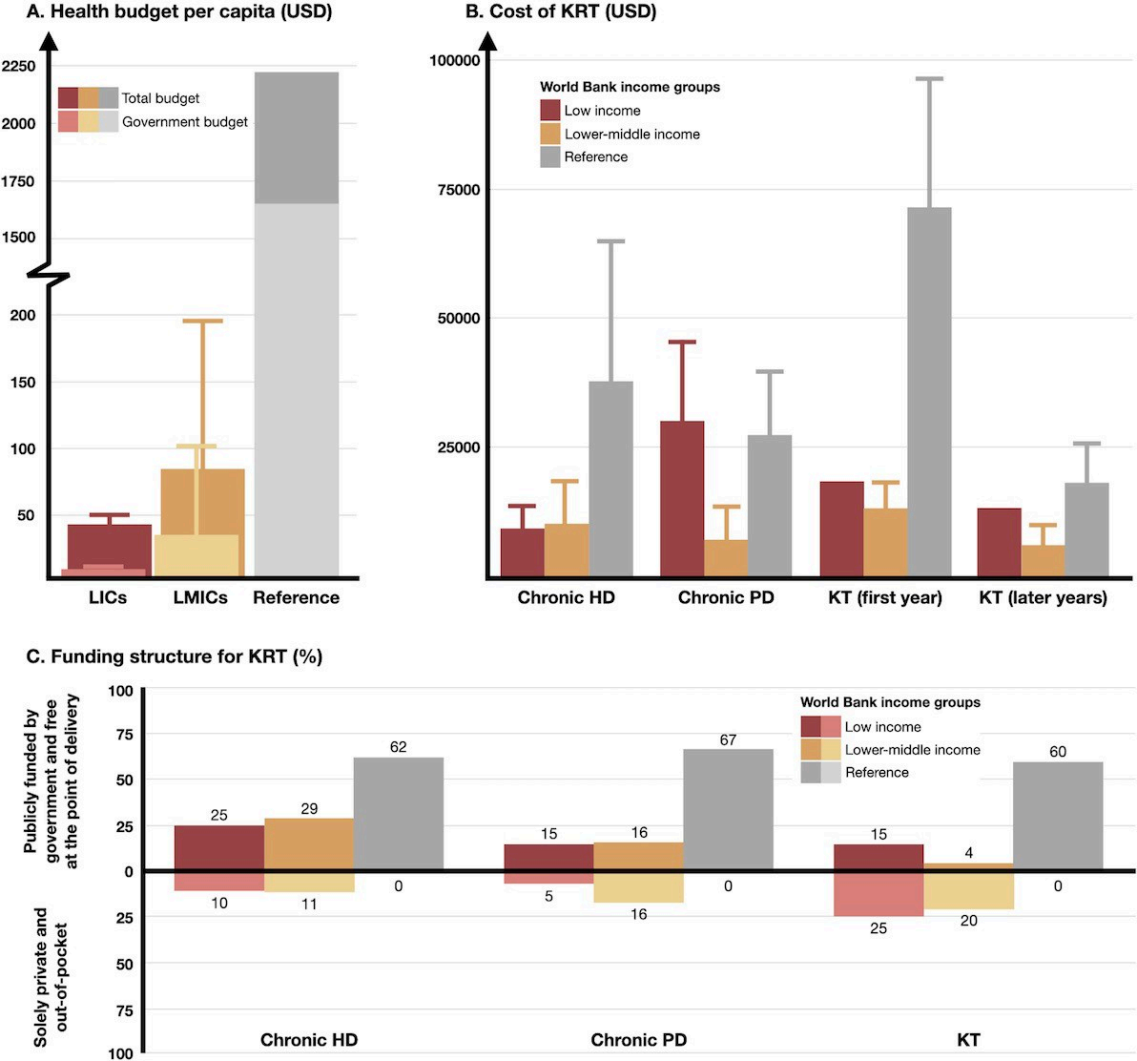
Total healthcare budget and funding for kidney replacement therapy in low- and lower-middle-income countries compared to high-income countries: (A) health budget, (B) cost of kidney replacement therapy, and (C) funding structures for KRT [12–99]. Abbreviations: HD = hemodialysis; KRT = kidney replacement therapy; KT = kidney transplantation; LICs = low-income countries; LMICs = lower-middle-income countries; PD = peritoneal dialysis; USD = United States Dollar.

Literature review data for health funding were available from 24 LICs and 43 LMICs. The median total and government health budgets per capita were US$43 and US$9 (21%) for LICs and US$85 and US$35 (41%) for LMICs, in contrast to US$2218 and US$1642 (74%) in HICs (Fig 1A and S2 Table) [12]. The median annual costs of HD were US$9064.8 and US$10,114.5 in LICs and LMICs, respectively, compared to US$37,685.4 for HICs. The median annual costs of PD were US$30,064.4 and US$7005.1 in LICs and LMICs, respectively, compared to US$27,206 for HICs. For KT, the median costs in the first year were US$18,269.1 and US$13,013.3 in LICs and LMICs, respectively, compared to US$71,445.6 for HICs. The median annual costs of KT in the later years were US$13,126.3 and US$5994 in LICs and LMICs, respectively, compared to US$17,996 for HICs (Fig 1B and S2 Table) [13–99].

The survey revealed that only 25% (n = 5) and 29% (n = 13) of LICs and LMICs, respectively, had chronic HD publicly funded by the government and provided free at the point of delivery, compared to 62% (n = 39) in HICs. Solely private and out-of-pocket funding for HD was reported in 10% (n = 2) of LICs and 11% (n = 5) of LMICs. For chronic PD, only 15% (n = 3) and 16% (n = 7) of LICs and LMICs, respectively, had government-funded care that was free at the point of delivery, contrasting with 67% (n = 42) in HICs. Solely private and out-of-pocket funding for HD was reported in 5% (n = 1) of LICs and 16% (n = 7) of LMICs. KT was funded via public system (government funding and free at the point of delivery) in 15% (n = 3) and 4% (n = 2) of LICs and LMICs, respectively, compared to 60% (n = 38) of HICs. Solely private and out-of-pocket funding for HD was reported in 25% (n = 5) of LICs and 20% (n = 9) of LMICs. In HICs, no solely private reimbursement was reported for HD, PD, or KT (Fig 1C and S2 Table).

### Availability of KRT

**Fig 2:**
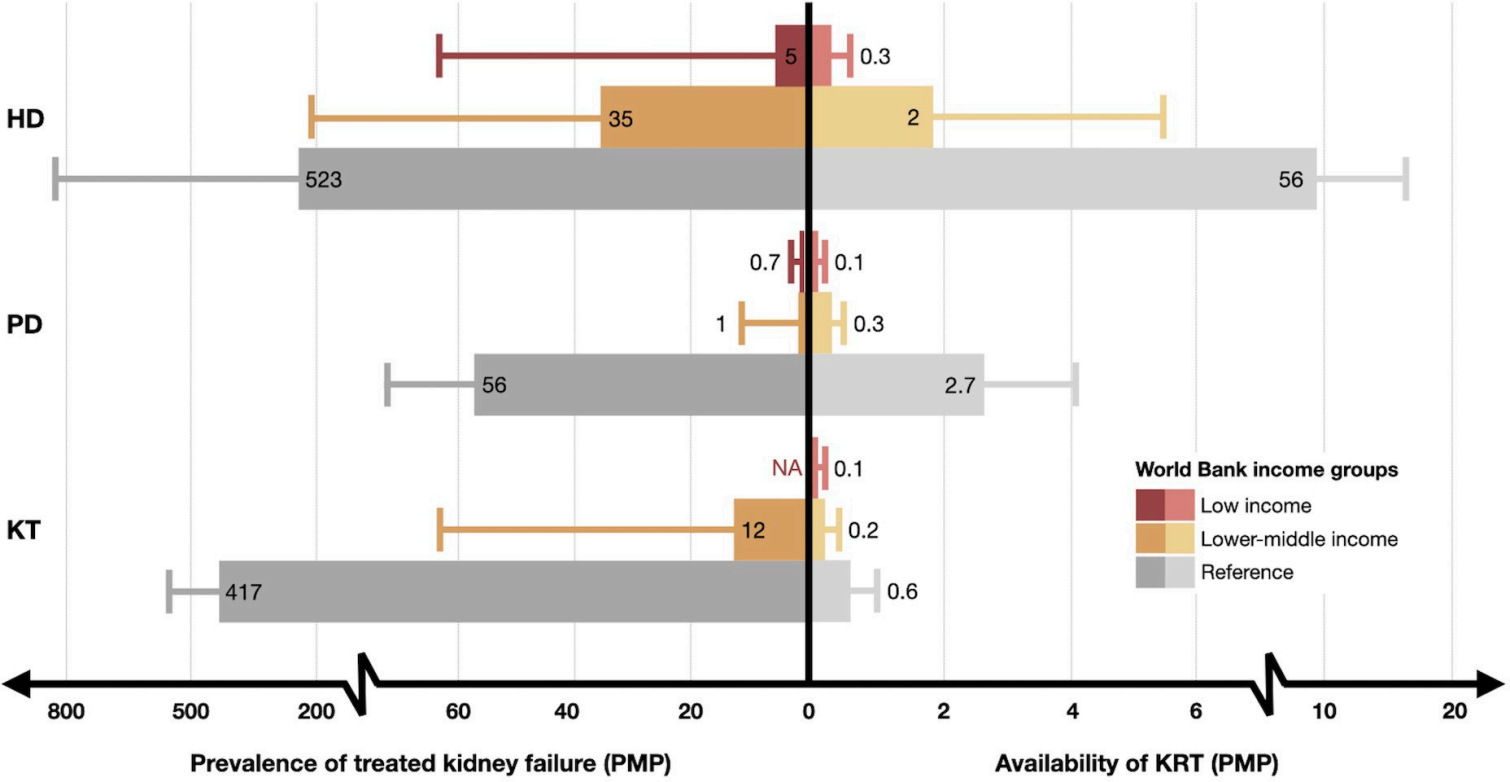
Prevalence of treated kidney failure and availability of kidney replacement therapy in low- and lower-middle-income countries compared to high-income countries [99–118]. Abbreviations: HD = hemodialysis; KRT = kidney replacement therapy; KT = kidney transplantation; NA = data not available; PD = peritoneal dialysis; PMP = per million population.

The prevalence of KRT revealed substantial disparities across LLMICs and HICs. The prevalence of chronic HD in LICs and LMICs was lower, with median values of 5.1 [1.2, 67.9] and 34.6 [10.1, 211.4] per million population (PMP), compared to 523.4 [355.7, 819.7] PMP in HICs. The prevalence of chronic PD also followed this pattern, with reported median values of 0.7 [0.0, 2.5] PMP in LICs, 1.3 [0.0, 10.8] PMP in LMICs, and 56.2 [37.9, 86.2] PMP in HICs. For KT, data were unavailable for LICs, while LMICs reported a median prevalence of 12 [5.5, 66.0] PMP, compared to 417 [279.0, 565.8] PMP in HICs (Fig 2 and S3 Table) [99–118].

Regarding the availability of HD centers, LICs and LMICs had median reported values of 0.3 [0.2, 0.6] PMP and 1.9 [0.8, 5.5] PMP, compared to 9.31 [4.8, 16.4] PMP in HICs. Chronic PD centers followed a similar pattern, with 0.1 [0.1, 0.2] PMP in LICs, 0.3 [0.1, 0.5] PMP in LMICs, and 2.7 [1.8, 4.1] PMP in HICs. For KT, the reported median availabilities were 0.1 [0.0, 0.2] PMP in LICs, 0.2 [0.1, 0.4] PMP in LMICs, and 0.6 [0.4, 1.0] PMP in HICs (Fig 2 and S3 Table).

### Accessibility and capacity of KRT

**Fig 3:**
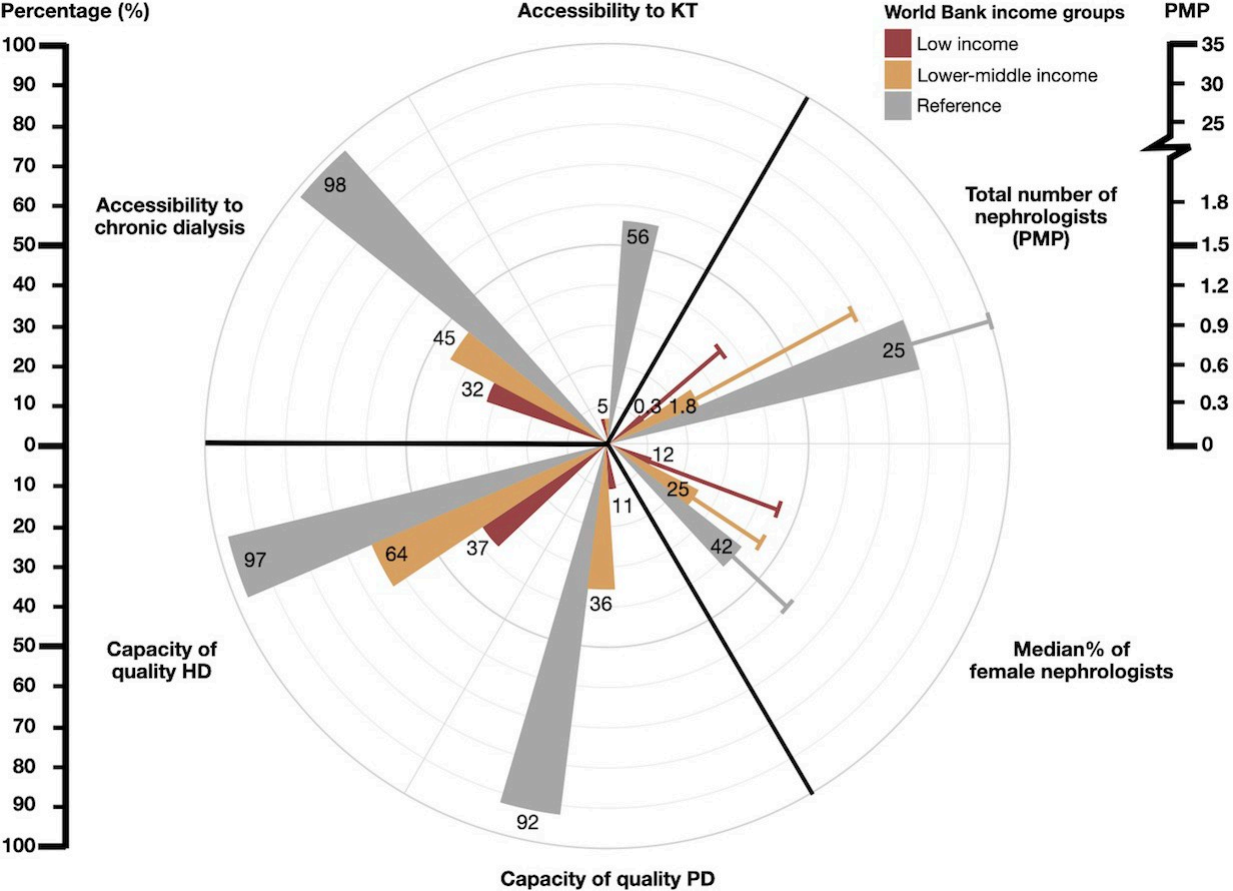
Capacity for kidney replacement therapy in low- and lower-middle-income countries compared to high-income countries. Abbreviations: HD = hemodialysis; KRT = kidney replacement therapy; KT = kidney transplantation; PD = peritoneal dialysis; PMP = per million population.

The accessibility to chronic dialysis, evaluated based on whether >50% of the national population could access KRT, showed variations, with 32% (n = 6) in LICs, 45% (n = 20) in LMICs, and 98% (n = 62) in HICs. Accessibility to KT was reported as 5% (n = 1) in LICs, 5% (n = 2) in LMICs, and 56% (n = 35) in HICs (Fig 3 and S3 Table). The variability of access to chronic dialysis showed a within-country variation in 61% (n = 11) in LICs, 54% (n = 22) in LMICs, and 11% (n = 7) in HICs. The impact of patient characteristics (age, gender, employment status) on access to chronic dialysis was reported in 33% (n = 6) of LICs, 39% (n = 16) of LMICs, and 8% (n = 5) of HICs. The variability in the access to KT also showed within-country variation in 14% (n = 1) in LICs, 37% (n = 10) in LMICs, and 16% (n = 10) in HICs. The variation in KT access influenced by patient characteristics was reported in 29% (n = 2) of LICs, 48% (n = 13) of LMICs, and 28% (n = 17) of HICs.

The capacity to provide quality chronic HD (3-4-hour HD session thrice weekly) was reported to be 37% in LICs and 64% in LMICs, compared to 97% in HICs. The capacity to provide quality chronic PD (defined as four daily exchanges on continuous ambulatory PD [CAPD] or equivalent cycles on automated PD) was reported as 11% in LICs and 36% in LMICs, compared to 92% in HICs.

## Workforce for kidney care

The median prevalence of nephrologists was 0.3 PMP in LICs, 1.8 PMP in LMICs, and 25.3 PMP in HICs. The percentages of nephrologists who were female were 12% in LICs, 25% in LMICs, and 42% in HICs (Fig 3). Significant shortages of nephrologists were reported in 90% of LICs, 80% of LMICs, and 51% of HICs. Transplant surgeon shortages were reported in 90% of LICs, 80% of LMICs, and 38% of HICs. Countries reported limitations in the availability of dialysis nurses, whereby shortages were reported in 70% of LICs, 64% of LMICs, and 52% of HICs. Dialysis technicians were reported as being in short supply in 65% of LICs, 64% of LMICs, and 22% of HICs. The availability of transplant coordinators was also reported to be limited in 80% of LICs, 76% of LMICs, and 25% of HICs. Dietitians were reported to be in short supply in 90% of LICs, 76% of LMICs, and 38% of HICs (S4 Table).

### Health information systems and structures for policies and advocacy for KRT provision

**Fig 4:**
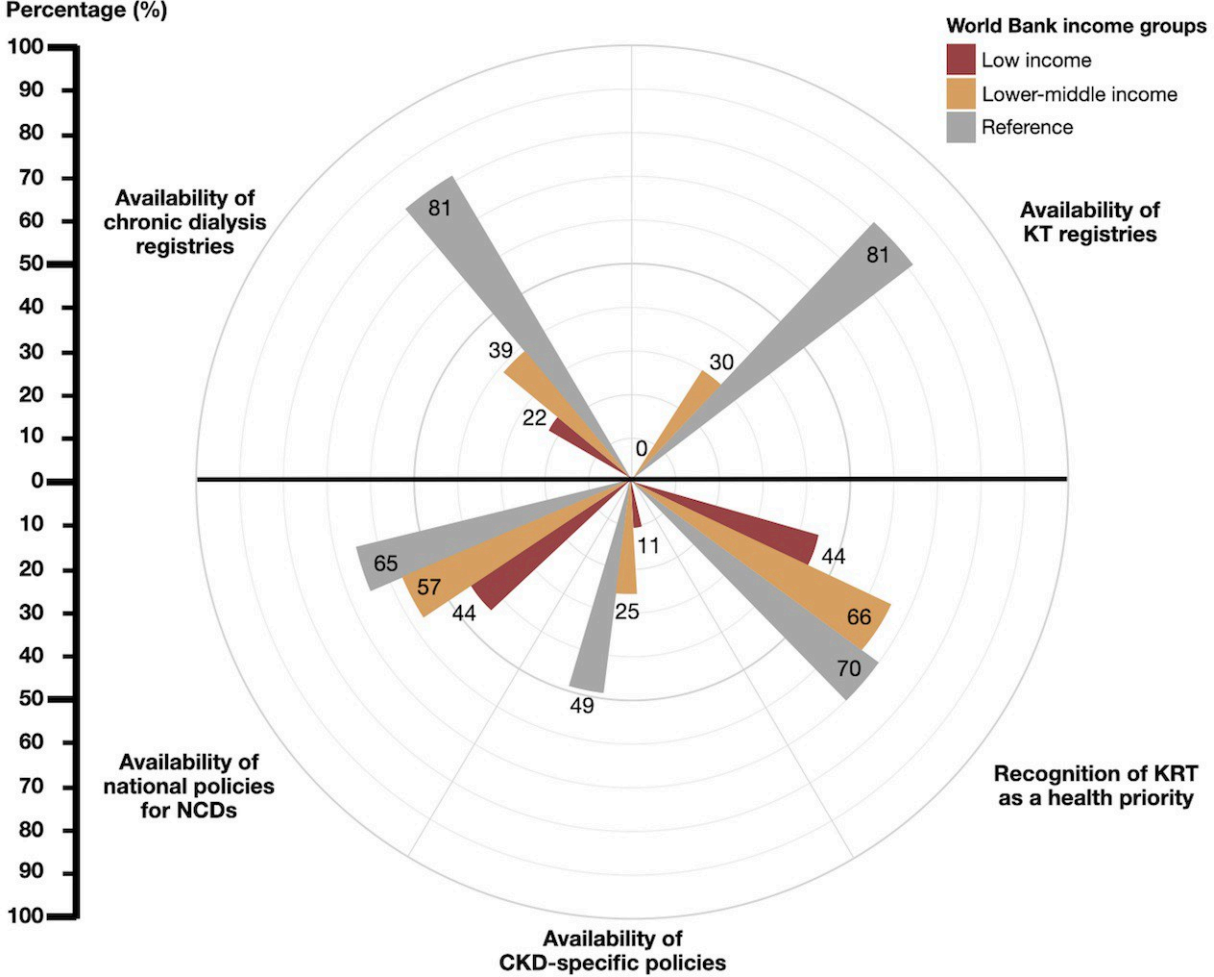
Registry, policy, and advocacy regarding kidney replacement therapy in low- and lower-middle-income countries compared to high-income countries. Abbreviations: CKD, chronic kidney disease; KRT, kidney replacement therapy; KT, kidney transplantation; NCD, non-communicable diseases.

Health information systems, including registries for chronic dialysis, were reported to be available in 22% of LICs and 39% of LMICs compared to 81% of HICs. KT registries were unavailable in all LICs and in 70% of LMICs compared to 20% of HICs. Regarding the availability of policies and advocacies for KRT, 44% of LICs reported national policies for NCDs, 11% had CKD-specific policies, and 44% recognized KRT as a health priority. In LMICs, these figures were 57%, 25%, and 66%, respectively. In contrast to HICs, 65% had national policies for NCDs, 49% had CKD-specific policies, and 70% recognized KRT as a health priority (Fig 4 and S3 Table).

## Discussion

This analysis revealed significant disparities in the organization of and structures for KRT provision in LICs and LMICs globally. There were significant gaps in funding, capacity for care (KRT centers) and distribution, accessibility to KRT, capacity for quality KRT, workforce, and health information systems for quality assurance and monitoring in KRT provision.

The major disparity we noted was in total healthcare financing (not restricted to KRT) with a substantially lower allocation to government health budgets in LICs, highlighting the under-investment these countries make in providing comprehensive healthcare, including KRT. This finding aligns with the previously reported barriers to universal health coverage, particularly in Africa and South Asia [119, 120]. The universal coverage model observed in HICs, frequently achieved through prepayment schemes, was consistent with our survey findings, indicating that none of the participating countries solely rely on out-of-pocket reimbursement across all KRT modalities [121]. However, implementing such an approach in LLMICs proves challenging due to variations in employment rates and financial security. Governments may consider cost-sharing or health insurance options to enhance access to quality care for people with kidney failure and mobilize funds [122, 123]. Nevertheless, while cost-sharing or health insurance options increase service availability, they could lead to negative health outcomes as some people may discontinue treatment or receive suboptimal care [124–128]. Considering that the annual cost per treated person in LLMICs significantly surpasses the average income [129], over-reliance on private reimbursement remains a significant barrier to expanding access to KRT services. While certain countries receive support from non-governmental organizations, these resources are not universally accessible [130, 131]. In addition, political conflict, instability, corruption, wars, and inflation may hinder effective budget allocation [132]. Notably, the median cost per capita for KRT provision was generally lower in the LLMICs compared to HICs, likely influenced by factors such as lower labor and infrastructure costs. Regulatory variations, pharmaceutical pricing, and limited use of technological advancements also impact costs. However, it is important to note that lower costs do not necessarily reflect the quality of care. Therefore, there is substantial potential for any incremental funding to enhance KRT provision in the LLMICs considerably.

In addition to healthcare financing, access to KRT was influenced by important geographic and patient-related factors. People residing in remote areas, women, older individuals, economically disadvantaged people, and those with higher severity of kidney failure face lower accessibility to KRT [133, 134]. Our findings indicated that the impact of patient characteristics on KRT accessibility is more pronounced in LLMICs, which often requires healthcare providers to make challenging decisions about which individuals will receive access to crucial kidney care [135]. The capacity to provide quality HD and PD in LLMICs remains suboptimal, resulting in inadequate dialysis services despite the availability of these modalities [63]. Moreover, although home-based dialysis (especially PD) could help to address rural transportation, it is infrequently available in LLMICs due to the high cost of PD fluids, largely due to the need to import consumables, insufficient PD training, challenges associated with PD outcomes, and a shortage of healthcare workforce [136]. Although it is well-established that home dialysis is cost-effective in HICs [137, 138], the data in LLMICs remain limited [139].

Another major barrier to optimal KRT provision is the availability and distribution of an effective workforce. LLMICs exhibited notably lower median prevalence of nephrologists and essential healthcare professionals for dialysis. The workforce shortage specifically related to KT, including transplant surgeons and coordinators, was reported to be higher. This suggests a potential deficit in specialized healthcare professionals crucial for delivering high-quality KRT services, especially KT. This is largely driven by limitations in working conditions and resources as well as opportunities for career growth hindering the retention of qualified workers [140]. Expanding nephrology training opportunities for healthcare providers and task shifting could contribute to the development of a stronger and more skilled nephrology workforce [7, 141, 142].

Additional challenges toward optimal and high-quality KRT provision in these settings is the lack of data monitoring systems. Kidney registries are crucial for developing a robust surveillance system for kidney care. In settings with limited resources, registries are pivotal in monitoring restricted modalities, such as dialysis and KT. They provide a comprehensive perspective on resource allocation and help identify areas for improvement [143, 144].

We have identified important gaps in care, and solutions are needed. Despite reported recognition of KRT as a health priority in LLMICs, national policies for NCDs and CKD-specific policies remain limited. Unlike UMICs or HICs, where innovative technologies enhance kidney care, LLMICs require comprehensive and cost-effective strategies that are practical in their context. This approach encompasses CKD prevention, early detection, and effective management to slow the progression to kidney failure [145, 146]. Public education and enhancing health literacy are crucial to empower people so that they can make informed health decisions [147]. International organizations, such as the Kidney Disease: Improving Global Outcomes (KDIGO) and the ISN, play a pivotal role in supporting LLMICs [148]. The ISN offers various programs to support workforce development, including fellowship grants, sister kidney center initiatives, and online teaching modules. The ISN’s SharE-RR Toolkit provides guidance on establishing registries to monitor kidney health, with special attention to the unique challenges in LLMICs. Collaboration at the global, regional, country (nephrology society), and individual levels should be prioritized to ensure the success of these initiatives [134, 149].

This analysis enriches the ISN-GKHA reports by providing a focused exploration exclusively on LLMICs, offering a comprehensive examination tailored to the unique healthcare challenges and landscapes of these regions. However, as a sub-analysis of the ISN-GKHA survey, it is based on cross-sectional data, capturing large-scale global trends but potentially lacking detailed insights at the individual country level. The analysis primarily adopts an economic lens, potentially overlooking important contextual factors such as human development index or demographic aspects. Incorporating these additional dimensions could provide a more nuanced understanding of the complex interactions influencing kidney health outcomes and disparities within LLMIC settings.

## Conclusions

We noted significant disparities in the organization, capacity, and structures for providing optimal KRT for people living with kidney failure in LLMICs, including workforce shortages, limited funding, and limited government responses (in terms of policies and strategies) toward prevention. Kidney diseases have a significant impact on population health and the economy. Given the burden of kidney failure and challenges in kidney care in LLMICs, there is a compelling need for the global nephrology community to persist in targeted efforts to close these gaps in care. Addressing these disparities will require collaborative efforts on multiple fronts, involving international, regional, and national stakeholder organizations, and meaningful public engagement.

## Supporting information

S1 Table List of participating low- and lower-middle-income countries by World Bank income groups and ISN regions. (DOCX)

S2 Table Global healthcare funding and cost of kidney replacement therapy in low- and lower-middle-income countries [12–99]. (DOCX)

S3 Table Organization and structures for kidney replacement therapy in low- and lower-middle-income countries. (DOCX)

S4 Table Workforce for kidney care in low- and lower-middle-income countries. (DOCX)

S1 Checklist Inclusivity in global research. (DOCX)

S1 Data (XLSX)
