# Supplementary material for: Landscape of kidney replacement therapy provision in low- and lower-middle income countries: A multinational study from the ISN-GKHA
Source: PLOS Glob Public Health. 2024 Dec 2;4(12):e0003979. doi: 10.1371/journal.pgph.0003979 (PMC11611141; doi:10.1371/journal.pgph.0003979)
Supplement: S1 Table — (DOCX) [file pgph.0003979.s001.docx]

**S1 Table. List of participating low- and lower-middle-income countries by World Bank income groups and ISN regions.**

| **World Bank income group** | **ISN region** | **Country** |
| --- | --- | --- |
| Low-income | Africa | Burkina Faso |
|  |  | Burundi |
|  |  | Central African Republic |
|  |  | Chad |
|  |  | Congo, Democratic Republic |
|  |  | Ethiopia |
|  |  | Gambia |
|  |  | Guinea |
|  |  | Madagascar |
|  |  | Malawi |
|  |  | Mali |
|  |  | Mozambique |
|  |  | Niger |
|  |  | Somalia |
|  |  | Sudan |
|  |  | Togo |
|  |  | Uganda |
|  |  | Zambia |
|  | Middle East | Syrian Arab Republic |
|  | South Asia | Afghanistan |
| Lower-middle-income | Africa | Angola |
|  |  | Benin |
|  |  | Cameroon |
|  |  | Cape Verde |
|  |  | Congo, Republic |
|  |  | Cote d'Ivoire |
|  |  | Egypt |
|  |  | Ghana |
|  |  | Kenya |
|  |  | Lesotho |
|  |  | Mauritania |
|  |  | Morocco |
|  |  | Nigeria |
|  |  | Senegal |
|  |  | Swaziland |
|  |  | Tanzania |
|  |  | Tunisia |
|  |  | Zimbabwe |
|  | Latin America | Bolivia |
|  |  | El Salvador |
|  |  | Haiti |
|  |  | Nicaragua |
|  | Middle East | Iran, Islamic Republic |
|  |  | Lebanon |
|  |  | West Bank and Gaza |
|  | Newly Independent States (NIS) and Russia | Kyrgyz Republic |
|  |  | Tajikistan |
|  |  | Ukraine |
|  |  | Uzbekistan |
|  | Oceania and South East Asia (OSEA) | Cambodia |
|  |  | Indonesia |
|  |  | Lao People’s Democratic Republic (PDR) |
|  |  | Myanmar |
|  |  | Papua New Guinea |
|  |  | Philippines |
|  |  | Samoa |
|  |  | Solomon Islands |
|  |  | Vanuatu |
|  |  | Vietnam |
|  | South Asia | Bangladesh |
|  |  | Bhutan |
|  |  | India |
|  |  | Nepal |
|  |  | Pakistan |
|  |  | Sri Lanka |
| High-income | Eastern and Central Europe | Croatia |
|  |  | Cyprus |
|  |  | Czech Republic |
|  |  | Estonia |
|  |  | Hungary |
|  |  | Latvia |
|  |  | Lithuania |
|  |  | Poland |
|  |  | Romania |
|  |  | Slovak Republic |
|  | Latin America | British Virgin Islands |
|  |  | Cayman Islands |
|  |  | Chile |
|  |  | Curaçao |
|  |  | Panama |
|  |  | Puerto Rico |
|  |  | Uruguay |
|  | Middle East | Kuwait |
|  |  | Oman |
|  |  | Qatar |
|  |  | Saudi Arabia |
|  |  | United Arab Emirates |
|  | North America and the Caribbean | Antigua and Barbuda |
|  |  | Aruba |
|  |  | Bahamas |
|  |  | Barbados |
|  |  | Bermuda |
|  |  | Canada |
|  |  | Trinidad and Tobago |
|  |  | Turks and Caicos Islands |
|  |  | United States |
|  | North and East Asia | Hong Kong |
|  |  | Japan |
|  |  | South Korea |
|  |  | Macao Special Administrative Republic (SAR), China |
|  |  | Taiwan |
|  | Oceania and South East Asia (OSEA) | Australia |
|  |  | Brunei Darussalam |
|  |  | New Caledonia |
|  |  | New Zealand |
|  |  | Singapore |
|  | Western Europe | Andorra |
|  |  | Austria |
|  |  | Belgium |
|  |  | Denmark |
|  |  | Finland |
|  |  | France |
|  |  | Germany |
|  |  | Greece |
|  |  | Iceland |
|  |  | Ireland |
|  |  | Israel |
|  |  | Italy |
|  |  | Liechtenstein |
|  |  | Luxembourg |
|  |  | Malta |
|  |  | Netherlands |
|  |  | Norway |
|  |  | Portugal |
|  |  | Spain |
|  |  | Sweden |
|  |  | Switzerland |
|  |  | United Kingdom |

Abbreviations: ISN = International Society of Nephrology
