# Supplementary material for: Landscape of kidney replacement therapy provision in low- and lower-middle income countries: A multinational study from the ISN-GKHA
Source: PLOS Glob Public Health. 2024 Dec 2;4(12):e0003979. doi: 10.1371/journal.pgph.0003979 (PMC11611141; doi:10.1371/journal.pgph.0003979)
Supplement: S2 Table — (DOCX) [file pgph.0003979.s002.docx]

**S2 Table. Global healthcare funding and cost of kidney replacement therapy in low- and lower-middle-income countries [12-99].**

|  | **LICs** | **LMICs** | **UMICs** | **HICs** |
| --- | --- | --- | --- | --- |
| **Health budgets per capita, median USD**^a^ **[IQR]** | | | | |
| Total health budget | 43 [30, 50] | 85 [57, 197] | 404 [299, 552] | 2218 [1230, 5105] |
| Government health budget | 9 [6, 10] | 35 [16, 103] | 247 [158, 350] | 1642 [817, 3998] |
| **Funding structure for KRT**^b^ | | | | |
| Participating countries, n | 20 | 45 | 39 | 63 |
| Publicly funded by government and free at the point of delivery, number of countries [N; (%)] | | | | |
| Chronic hemodialysis | 5 (25) | 13 (29) | 17 (45) | 39 (62) |
| Chronic peritoneal dialysis | 3 (15) | 7 (16) | 17 (45) | 42 (67) |
| Medications for kidney transplantation | 3 (15) | 2 (4) | 16 (42) | 38 (60) |
| Solely private and out-of-pocket, number of country (%) | | | | |
| Chronic hemodialysis | 2 (10) | 5 (11) | 0 (0) | 0 (0) |
| Chronic peritoneal dialysis | 1 (5) | 7 (16) | 1 (3) | 0 (0) |
| Medications for kidney transplantation | 5 (25) | 9 (20) | 1 (3) | 0 (0) |
| **Annual cost of KRT, median USD**^a^ **[IQR]** | | | | |
| Chronic hemodialysis^c^ | 9064.8  [4185.4, 13792.5] | 10114.5  [5205.4, 18659.5] | 14871.8 [10344.4, 17622.5] | 37685.4  [23981.6, 65119.9] |
| Chronic peritoneal dialysis^d^ | 30064.4  [14557.5, 45571.3] | 7005.1  [5205.4, 13952.7] | 14473.9  [8067.7, 21312.1] | 27206.0  [18780.1, 39825.9] |
| Kidney transplantation (first year) | 18269.1  [18269.1, 18269.1] | 13013.3  [5720.5, 18399.8] | 22301.9 [15598.6, 29780.3] | 71445.6  [41108.3, 96430.4] |
| Kidney transplantation (later years) | 13126.3  [13126.3, 13126.3] | 5994.0  [3307.1, 9665.5] | 14594.8 [4922.3, 15599.2] | 17996.0  [10630.2, 25242.0] |
| ^a^USD in the year 2021. ^b^Survey response data.  ^c^Data represents cost of chronic in-center hemodialysis per person. ^d^Data represents cost of chronic continuous ambulatory peritoneal dialysis per person. Abbreviations: IQR= interquartile range; KRT = kidney replacement therapy; LICs = low-income countries; LMICs = lower-middle-income countries; PMP = per million population; USD = United States Dollar. | | | | |
