# Supplementary material for: Landscape of kidney replacement therapy provision in low- and lower-middle income countries: A multinational study from the ISN-GKHA
Source: PLOS Glob Public Health. 2024 Dec 2;4(12):e0003979. doi: 10.1371/journal.pgph.0003979 (PMC11611141; doi:10.1371/journal.pgph.0003979)
Supplement: S3 Table — (DOCX) [file pgph.0003979.s003.docx]

**S3 Table. Organization and structures for kidney replacement therapy in low- and lower-middle-income countries.**

|  | **LICs** | **LMICs** | **UMICs** | **HICs** |
| --- | --- | --- | --- | --- |
| **Prevalence of treated kidney failure, median PMP [IQR]*** | | | | |
| Chronic hemodialysis | 5.1  [1.2, 67.9] | 34.6  [10.1, 211.4] | 330.5 [177.3, 654.3] | 523.4  [255.7, 819.7] |
| Chronic peritoneal dialysis | 0.7 [0.0, 2.5] | 1.3 [0.0, 10.8] | 17.9 [9.3, 52] | 56.2 [37.9, 86.2] |
| Kidney transplantation | NA | 12 [5.5, 66.0] | 83 [47.0, 137.1] | 417 [279.0, 565.8] |
| **Participating countries, n** | 19 | 45 | 38 | 63 |
| **Availability of KRT centers, median PMP [IQR]** | | | | |
| Chronic in-center hemodialysis | 0.3 [0.2, 0.6] | 1.9 [0.8, 5.5] | 5.4 [3.9, 10.0] | 9.3 [4.8, 16.4] |
| Chronic peritoneal dialysis | 0.1 [0.1, 0.2] | 0.3 [0.1, 0.5] | 1.1 [0.6, 1.9] | 2.7 [1.8, 4.1] |
| Kidney transplantation | 0.1 [0.0, 0.2] | 0.2 [0.1, 0.4] | 0.4 [0.3, 0.7] | 0.6 [0.4, 1.0] |
| **Accessibility to KRT, n (%)** | | | | |
| Chronic dialysis | 6 (32) | 20 (45) | 33 (87) | 62 (98) |
| Depending on different part of the country | 11 (61) | 22 (54) | 16 (43) | 7 (11) |
| Depending on patient characteristics^a^ | 6 (33) | 16 (39) | 7 (19) | 5 (8) |
| Kidney transplantation | 1 (5) | 2 (5) | 10 (26) | 35 (56) |
| Depending on different part of the country | 1 (14) | 10 (37) | 9 (27) | 10 (16) |
| Depending on patient characteristics^a^ | 2 (29) | 13 (48) | 9 (27) | 17 (28) |
| **Capacity of quality KRT, n (%)** |  |  |  |  |
| Chronic hemodialysis^b^ | 7 (37) | 29 (64) | 37 (97) | 61 (97) |
| Chronic peritoneal dialysis^c^ | 2 (11) | 16 (36) | 25 (66) | 58 (92) |
| **Health information system (registries), n (%)** | | | | |
| Chronic dialysis | 4 (22) | 17 (39) | 30 (81) | 51 (81) |
| Kidney transplantation | 0 (0) | 13 (30) | 30 (81) | 51 (81) |
| **Availability of policies and advocacies for KRT, n (%)** | | | | |
| National policies for NCDs | 8 (44) | 25 (57) | 17 (46) | 41 (65) |
| CKD-specific policies | 2 (11) | 11 (25) | 16 (43) | 31 (49) |
| Recognition of KRT as a health priority | 8 (44) | 29 (66) | 21 (57) | 44 (70) |
| * References #99-118  ^a^Patient characteristics included age, gender, and employment status. ^b^Number of countries with >50% available hemodialysis with the capacity for a 3-4 hour HD session thrice weekly. ^c^Number of countries with >50% available peritoneal dialysis with the capacity for daily four exchanges on continuous ambulatory peritoneal dialysis or equivalent dose on automated peritoneal dialysis. Abbreviations: CKD = chronic kidney disease; IQR = interquartile range; KRT = kidney replacement therapy; LICs = low-income countries; LMICs = lower-middle-income countries; NA = data not available; NCD = non-communicable diseases; PMP = per million population. | | | | |
