# Supplementary material for: Landscape of kidney replacement therapy provision in low- and lower-middle income countries: A multinational study from the ISN-GKHA
Source: PLOS Glob Public Health. 2024 Dec 2;4(12):e0003979. doi: 10.1371/journal.pgph.0003979 (PMC11611141; doi:10.1371/journal.pgph.0003979)
Supplement: S4 Table — (DOCX) [file pgph.0003979.s004.docx]

**S4 Table. Workforce for kidney care** **in low- and lower-middle-income countries.**

|  | **LICs** | **LMICs** | **UMICs** | **HICs** |  |  |
| --- | --- | --- | --- | --- | --- | --- |
| **Participating countries, N** | 20 | 45 | 39 | 63 |  |  |
| **Total number of workforces** | | | | | | |
| Nephrologists, median PMP [IQR] | 0.3 [0.2, 1.1] | 1.8 [0.6, 4.9] | 11.9 [5.9, 18.4] | 25.3 [18.0, 35.4] |  |  |
| Female nephrologists, median % [IQR] | 12 [10, 46] | 25 [10, 46] | 40 [25, 55] | 42 [30, 60] |  |  |
| **Shortage of workforce, N (%)** | |  |  |  |  |  |
| Nephrologists | 18 (90) | 36 (80) | 21 (54) | 32 (51) |  |  |
| Transplant surgeons | 18 (90) | 36 (80) | 31 (79) | 24 (38) |  |  |
| Dialysis nurses | 14 (70) | 29 (64) | 21 (54) | 33 (52) |  |  |
| Dialysis technicians | 13 (65) | 29 (64) | 18 (46) | 14 (22) |  |  |
| Transplant coordinators | 16 (80) | 34 (76) | 25 (64) | 16 (25) |  |  |
| Dietitians | 18 (90) | 34 (76) | 26 (67) | 24 (38) |  |  |
| Abbreviations: IQR = interquartile range; KRT = kidney replacement therapy; LICs = low-income countries; LMICs = lower-middle-income countries; PMP = per million population. | | | | | |  |
